# Cultural adaptation and psychometric properties of the Jefferson empathy scale health professions students’ version in SpanishOccupational therapy students

**DOI:** 10.1186/s12909-021-02845-y

**Published:** 2021-09-06

**Authors:** Sergio Serrada-Tejeda, Patricia Sánchez-Herrera-Baeza, Mª. Pilar Rodríguez-Pérez, Nuria Máximo-Bocanegra, Rosa Mª. Martínez-Piédrola, Nuria Trugeda-Pedrajo, Elisabet Huertas-Hoyas, Marta Pérez-de-Heredia-Torres

**Affiliations:** Department of Physical Therapy, Occupational Therapy, Rehabilitation and Physical Medicine, Rey Juan Carlos University, Avenida de Atenas s/n. CP.28922, Alcorcón, Madrid, Spain

**Keywords:** Empathy, Assessment, Occupational therapy, Students, Psychometrics

## Abstract

**Background:**

In occupational therapy, empathy is a fundamental concept and has a positive impact on health and quality of care outcomes for patients. It is a basic and essential concept that should prevail in the training of occupational therapy students. The aim of this study is to validate and cross-culturally adapt the Jefferson Medical Empathy Scale, version for health professionals (JSE-HPS) in a sample of Spanish university students of occupational therapy.

**Methods:**

A cross-sectional descriptive study was conducted between 2019 and 2020. A convenience sample was selected, consisting of 221 students from the four courses of the Occupational Therapy degree at the Universidad Rey Juan Carlos during the 2019–20 academic year. Each of the participants voluntarily and anonymously completed a sociodemographic data sheet (including age and sex), in addition to the following assessment scales: JSE-HPS and the Interpersonal Reactivity Index (IRI).

**Results:**

A culturally adapted version of the JSE-HPS that guarantees conceptual and grammatical equivalence specific to the study population was obtained. The psychometric analysis of the translated version showed a Cronbach coefficient α of 0.786. The test-retest reliability analysis showed an intraclass correlation coefficient of 0.90 (95% CI = 0.86–0.93, *p* < 0.0001). Confirmatory factor analysis (CFA) showed positive results (χ^2^ = 269.095, df = 167, *p* < 0.001, Confirmatory Fit Index [CFI] = 0.90, Root Mean Square Error of Approximation [RMSEA] = 0.04).

**Conclusion:**

The cultural adaptation and psychometric results suggest that the Spanish version of the JSE-HPS is a valid and reliable way to evaluate the empathic ability of occupational therapy students.

## Background

The term empathy, which derives from the Greek word “empatheia”, has been originally conceptualized as a capacity that enables putting ourselves in the place of others by appreciating their perspective as well as perceiving their emotions. This conceptualization has remained constant until the 1960s, when different researchers start considering empathy as a combined result of cognitive factors, such as understanding and objectivity, and emotional factors, such as feelings and subjectivity. From a cognitive perspective, authors such as Hojat et al. [1, 2], conceive empathy as understanding the mental state of another person using cognitive processes of oneself, allowing one to understand the way others perceive the world. On the other hand, the emotional approach is based on the ability to experience an appropriate emotional response as a result of emotional state and feelings of other people, generating a shared affection or vicarious feeling [3]. Because of this combined conceptualization, the assessment of empathy has determined that cognitive factors are more dependent on cultural aspects and learning, while emotional aspects are considered as an innate aspect [4].

Considering this combined perspective, empathy has been used in certain disciplines to explain social interactions and, historically, it has been a concept studied mainly in the field of psychology. However, due to the need to capture the essence and measure empathic attitude within the framework of patient care, the Jefferson Scale of Physician Empathy (JSPE) was developed by Hojat et al. [5]. In this context, empathy was considered as a cognitive attribute, that involves the ability to understand a patient’s internal experiences and perspective, combined with the ability to communicate this understanding [6]. From this perspective, the JSPE, considered empathy as a cognitive attribute that allows understanding of emotions, experiences, and perspectives of patients, but also understanding of emotions to be communicated effectively.

This point of view has been supported by other authors, such as Bylund and Makoyl [7], who highlighted the importance of communication in the understanding of the patient, supporting the idea that empathy is an element in which the cognitive and emotional components are not completely independent. Similarly, other authors [8–10] considered empathic relationships with patients to be a type of significant interpersonal connection for health professionals, and these connections can serve as a buffer against job dissatisfaction, burnout, and work-related stress.

In health care oriented with patient care, recognizing, and searching for occasions in which to show empathic behavior is a central element of the health professional-patient relationship [5, 11]. In recent years, empathy has acquired greater interest in the field of health education due to recent studies published that showed a decrease in empathy scores among medical professionals (Chen et al., 2017) and in other health professions, such as nursing or dentistry [12] due to stressful clinical experiences [13–16]. This results have been also observed by Brown et al. [17] on first-year occupational therapy students, where it was identified that, although the level of empathy was similar to that observed in students of other health disciplines, the scores obtained were not as high as those observed in similar studies, suggesting that the first year of university training does not impact the level of empathy of the occupational therapy student.

However, in the field of occupational therapy, the ability to empathize with patients during their recovery process is essential to be able to cope and provide the necessary support and understanding for the difficulties that may arise as a result of an alteration in occupational performance. Therefore, in order to achieve meaningful therapeutic outcomes for each individual, during the intervention process, the occupational therapist must pay special attention to the variety of patient roles and contexts, directing the process towards the achievement of relevant goals and promoting empathic communication focused on facilitating patient understanding.

However, although empathy may be impaired due to lack of good mentors, lack of time and recognition, or the increased use of diagnostic technology in the healthcare setting (Brown et al. [17], its assessment is indispensable in the health professions educational context because the development of empathic attitudes has been found to improve patient health outcomes [18]. In these educational contexts, the JSE-HPS [3] is the most widely used scale to assess empathy which has been also adapted and translated to multiple contexts and has been used in different settings, showing evidence of validity in patient outcomes, clinical competence and personality measurement, as well as evidence comparing groups (i.e., gender). Nevertheless, checking the adequate adjustment of the translated version in the study context requires a validation process to verify and ensure the interpretability of the results, and although the JSE-HPS version has been widely used for assessment with different types of student populations such as nurses, physical therapists and pharmacists [19–24], and has been translated into other languages, such as Italian, Japanese or Finnish [21], not all versions have been culturally adapted, nor have the psychometric properties of the resulting versions been analyzed.

Therefore, given that there are currently no adapted and culturally validated versions in Spanish for occupational therapy students, we believe in the need for a version of the JSE-HPS that allows us to explore this aspect in occupational therapy students since empathy is a skill that facilitates and supports the understanding and development of the therapeutic health professional-patient relationship. For these reasons this study aims to:

- translate and culturally adapt the JSE-HPS in a sample of Spanish university students of occupational therapy,
- analyze the psychometric properties of the JSE-HPS and compare the results with other works on the analysis of empathy in students of other health professions, and
- explore and analyze the relationship between empathy and the Interpersonal Reactivity Index (IRI) to establish criterion validity.

## Methodology

A cross-sectional descriptive study was conducted between 2019 and 2020 to culturally adapt and analyze the psychometric properties of the Spanish version of the JSE-HPS in university occupational therapy students. A convenience sample was selected, consisting of the students of the four courses of the degree of Occupational Therapy at the Universidad Rey Juan Carlos during the academic year 2019–20. Inclusion criteria of the study were (1) students of occupational therapy at the Universidad Rey Juan Carlos and (2) age over 18 years. The students of the four courses have received the same curricular training and the pedagogical approach has been the same, as there have been no changes or restructuring of the four-years-training itinerary. Their training varies according to the academic year they take, focusing on theoretical and medical training during the first 2 years, and then, during the last 2 years, on specific curricular training in occupational therapy intervention methods as well as clinical practices.

To provide adequate precision and power of the parameter estimates and indexes of model fit, according to Kline [25] and based on study reviews [26] setting a minimum sample size in confirmatory factor analysis (CFA) of *N* = 200 is necessary. This suggestion is also consistent with the current literature (Schumacher & Lomax, 2010) and general rules of thumb, such as the ratio of the number of people (N) to the number of measured variables (p),) i.e. N > p [27] ranging from 5 with a minimum *N* > 100 ([28], cited in [27]), to 10; or the number of cases (N) to the number of estimated parameters (q) i.e. N:q, which considers that for CFA can range from 5 to 10 cases [29–32]. Finally, a sample of volunteer participants of 221 students was organized. Each of the participants anonymously completed a sociodemographic data sheet (age and sex), in addition to the following assessment scales:

- Jefferson’s Medical Empathy Scale, Healthcare Professional Version (JSE-HPS [2]): consists of 20 items that are scored on a Likert response from 1 (strongly disagree) to 7 (strongly agree). Ten of these items are worded positively and the other ten are worded negatively to avoid social desirability, approval, and acquiescence in the answers. The scale varies from 20 to 140 points, with higher scores indicating greater empathic orientation.
- Davis Interpersonal Reactivity Index (IRI [33]): This scale is especially useful in the research of the multidimensionality of the empathic process in the general population. This scale is adapted to Spanish (Mestre et al., 2002) and consists of 28 items distributed in four subscales that measure four dimensions of the integrative concept of empathy: perspective taking, fantasy, empathic concern and personal distress or discomfort.

### Ethical approval

This study was approved by the Research Ethics Committee of the Rey Juan Carlos University with number 0504201907319. All methods were performed in accordance with the relevant guidelines and regulations. Informed consent to participate as well as written consent for anonymous data collection were provided.

### Procedures

#### Cultural adaptation

The implementation of cultural adaptation was approved by Thomas Jefferson University (Center for Research in Medical Education and Health Care). For this phase, the linguistic criteria developed by The International Test Commission [34] and Hambleton and Li [35] were taken into consideration, developing three different phases: direct and reverse translation, review by a linguistic expert and panel expert review.

For the direct translation phase, a bilingual occupational therapist and a scientific translator independently translated the scale. Once the different translations were obtained, the research team reviewed each of the translations, identifying possible discrepancies with the original version and finally producing a first draft. This first version was then sent to the back-translation team, made up of two different scientific translators, who following a blinded process, translated the first version into English. Once the second version of the questionnaire was obtained, a panel of experts analyze and compare its conceptual equivalence with the original questionnaire, finally obtaining the preliminary version. This preliminary version was reviewed by a linguistic expert, who analyzed the semantic and grammatical adequacy of the terms used, ensuring the comprehensibility of the version in the context and target population. Table 1 shows some examples of the items that underwent modifications after the cultural adaptation process.

**Table 1:** Examples of direct/back translation procedures

Original Test Item	Spanish Translation	Recommended modifications	Final translation
Asking patients about what is happening in their personal lives is not helpful in understanding their physical complaints	Preguntar a los pacientes sobre lo que ocurre en su vida personal no es útil para comprender sus quejas físicas	Include the term *“dolencias”*	Preguntar a los pacientes sobre lo que ocurre en su vida personal no es útil para comprender sus quejas/dolencias físicas
It is difficult for a physician to view things from patients’ perspectives	Es difícil para un profesional sanitario ver las cosas desde el punto de vista de los pacientes	Replace*“el punto de vista”* for *“perspectiva”*	Es difícil para un profesional sanitario ver las cosas desde la perspectiva de los pacientes
Attentiveness to patients’ personal experiences does not influence treatment outcomes	Prestar atención a las experiencias personales de los pacientes no influye en los resultados de la intervención	Replace*“intervención”* for *“tratamiento”*	Prestar atención a las experiencias personales de los pacientes no influye en los resultados del tratamiento
Physicians should not allow themselves to be influenced by strong personal bonds between their patients and their family members	Los profesionales sanitarios no deberían permitir verse influidos por los fuertes vínculos que se establecen con sus pacientes y miembros de la familia	Rephrase the sentence and replace the term *“miembros de la familia”* for *“familiares”*	Los profesionales sanitarios no deberían permitirse verse influidos por los fuertes vínculos establecidos con sus pacientes y familiares

#### Analysis of psychometric properties

Analysis of the variables was performed using the IBM SPSS statistical program for Windows, version 22.0 and the IBM SPPS Amos, version 23.0 (IBM Corp., Armonk, NY, USA) for the CFA.

Construct validity: in order to investigate whether the factor structure identified by the authors of the original questionnaire can be replicated in the new dataset from 221 participants, confirmatory factor analysis (CFA) was conducted. For this purpose, measures of the model’s goodness of fit were assessed through the absolute fit measures: the Chi-square divided by degrees of freedom (CMIN/DF), the Root Mean Square Error of Approximation (RMSEA), and incremental adjustment measures such as the comparative fit index (CFI). In general threshold values of less than 0.05 for RMSEA are indicative of good fit of the model in relation to the degrees of freedom (Schermelleh-Engel et al., 2003 [36];). CMIN/DF < 3 indicates an acceptable fit between hypothetical model and sample data [37] and CFI > 0.85 indicate good levels of fit between data and model [38–40].

Reliability: to analyze the internal consistency of the scale, the Cronbach coefficient α was obtained. Cronbach’s alpha values > 0.70 were considered acceptable to guarantee the internal consistency of the questionnaire [41]. In addition, item-total correlations and intraclass correlation coefficient (ICC) were examined. Test-retest reliability was analyzed in a sample of 60 volunteer participants, who were randomly assigned and completed the scale 15 days after the first administration.

Convergent validity: determined by analyzing the relationship between JSE-HPS scores with those of another scale used as the gold standard for measuring empathy. In this case, the IRI was used in a way similar to that carried out by the authors of the original scale.

## Results

The present study included a final sample of 221 participants, of which 88.2% were women (*N* = 195) and 11.8% were men (*N* = 26). The average age of the total sample was 20.62 (SD = 2.7) years. The socio-demographic data of the sample are indicated in Table 2.

**Table 2:** Sample descriptive results

	Total sample (***N*** = 221)	Men	Women
Sex (n, %)		26 (11.8)	195 (88.2)
Age [mean (SD)]	18–47 [20.62 (2.7)]	20.58 (1.62)	20.63 (2.88)
Jefferson Total [mean (SD)]	122.28 (8.42)	112.92 (10.2)	123.53 (7.33)
Dimension 1 (PT)	64.96 (4.16)	60.12 (6.25)	65.61 (3.33)
Dimension 2 (CC)	48.04 (4.54)	44.54 (5.5)	48.51 (4.2)
Dimension 3 (SPS)	9.25 (2.41)	8.27 (1.37)	9.38 (2.49)
IRI Total [mean (SD)]	69.84 (9.80)	64.50 (7.72)	70.55 (9.89)
Dimension 1 (PT)	18.28 (3.37)	18.23 (3.12)	18.29 (3.41)
Dimension 2 (FS)	18.69 (5.07)	16.08 (3.77)	19.04 (5.13)
Dimension 3 (EC)	22.02 (3.34)	18.96 (2.93)	22.43 (3.18)
Dimension 4 PD	10.85 (3.36)	11.23 (2.98)	10.79 (3.41)

Note: *PT* (Perspective Taking); *CC* (Compasionate Care); *SPS* (Standing in Patient’s Shoes); *FS* (Fantasy); *EC* (empathic Concern); *PD* (Personal Distress)

### Construct validity

**Fig. 1:**
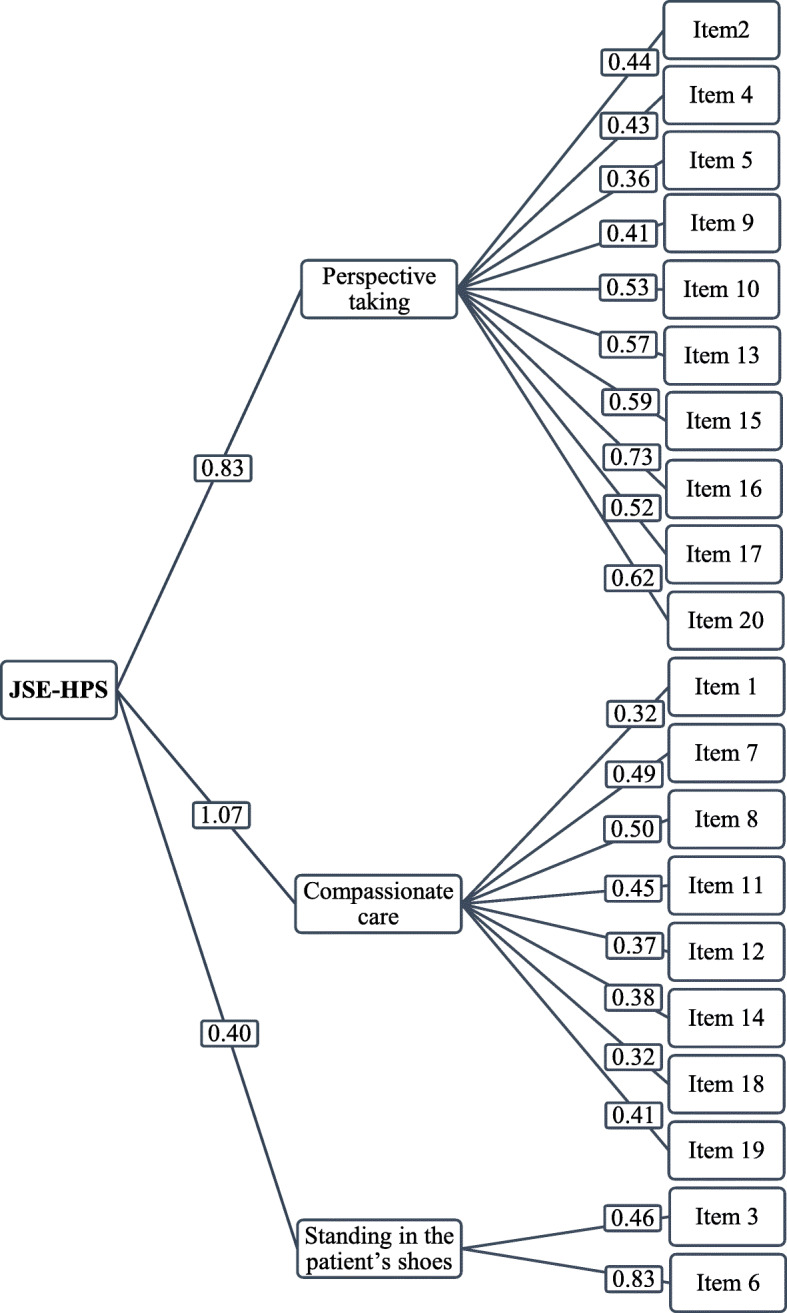
Model for the confirmatory factor analysis

Using CFA, the construct validity was verified with the factor model proposed by the authors of the original questionnaire conforming to the data that we have obtained in the occupational therapy students. All items obtained factor loads greater than 0.3 (Fig. 1) and the resulting model had an acceptable fit (χ^2^ = 269,095, df = 167, *p* < 0.001; CFI = 0.87; RMSEA = 0.04). The CFA with Amos Sotfware confirmed the three-factor structure for the JSE-HS, and its composition, psychometric properties and factor loading are shown in Table 3. The three JSE-HPS factors explain 42.98% of the variance. The first factor, perspective taking, was the most important factor because it evaluates the cognitive element of empathy. This factor grouped ten items and explained 21.34% of the variance. The second factor, attention with compassion, evaluated the emotional dimension of empathy and included six items that explained 13.05% of the variance. Finally, the third factor, putting oneself in the patient’s place, evaluated emotional attachment, included two items, and explained 12.05% of the variance.

**Table 3:** Factor loading for the 20 items of the JSE-HPS questionnaire

Ítem	Descripción del ítem	Factor I	Factor II	Factor III	
20	I believe that empathy is an important factor in patients’ treatment	.696			Eigenvalue:2.846 Cronbach’s α: 0.780 IC 95% [0.734–0.821]
13	Health care providers should try to understand what is going on in their patients’ minds by paying attention to their non-verbal cues and body language	.662			
16	Health care providers’ understanding of the emotional status of their patients. as well as that of their families is one important component of the health care provider – patient relationship	.625			
17	Health care providers should try to think like their patients in order to render better care	.602			
15	Empathy is a therapeutic skill without which a health care provider’s success is limited	.596			
4	Understanding body language is as important as verbal communication in health care provider - patient relationships	.567			
10	Patients value a health care provider’s understanding of their feelings which is therapeutic in its own right	.547			
2	Patients feel better when their health care providers understand their feelings	.454			
9	Health care providers should try to stand in their patients’ shoes when providing care to them	.390			
5	A health care provider’s sense of humor contributes to a better clinical outcome	.387			
7	Attention to patients’ emotions is not important in patient interview		.684		Eigenvalue: 1.455 Cronbach’s α: 0.720 IC 95% [0.702–0.798]
1	Health care providers’ understanding of their patients’ feelings and the feelings of their patients’ families does not influence treatment outcomes		.661		
8	Attentiveness to patients’ personal experiences does not influence treatment outcomes		.635		
11	Patients’ illnesses can be cured only by targeted treatment; therefore. Health care providers’ emotional ties with their patients do not have a significant influence in treatment outcomes		.528		
12	Asking patients about what is happening in their personal lives is not helpful in understanding their physical complaints		.507		
19	I do not enjoy reading non-medical literature or the arts		.519		
14	I believe that emotion has no place in the treatment of medical illness		.370		
18	Health care providers should not allow themselves to be influenced by strong personal bonds between their patients and their family members		.369		
3	It is difficult for a health care provider to view things from patients’ perspectives			.792	Eigenvalue: 1.396 Cronbach’s α: 0.698 IC 95% [0.696–0.720]
6	Because people are different. it is difficult to see things from patients’ perspectives			.624	

Note: Factor 1: perspective taking; factor 2: compassionate care; factor 3: standing in the patient’s shoes

Items are listed by their factor loadings size within each factor. Items were scored based on a seven-point Liker scale from 1 (strongly disagree) to 7 (strongly agree) except reverse-scored items (items 1, 3, 6, 7, 8, 11, 12, 14, 18 and 19)

### Reliability

The scale obtained a Cronbach coefficient α of 0.786. The test-retest reliability analysis showed an intraclass correlation coefficient of 0.90 (95% CI = 0.86–0.93, *p* < 0.0001). Pearson’s item-total correlation coefficients ranged from 0.34–0.51 and were all statistically significant (*p* < 0.01) (Table 4).

**Table 4:** Mean, standard deviation and item-total correlation

Item	Item description	Mean	SD	Item-total
1	La comprensión del profesional sanitario de los sentimientos de los pacientes y de los familiares no influye en los resultados del tratamiento	6.13	1.45	.488**
2	Los pacientes se sienten mejor cuando el profesional sanitario comprende sus sentimientos	6.75	.501	.391**
3	Es difícil para un profesional sanitario ver las cosas desde la perspectiva de los pacientes	4.66	1.378	.378**
4	Comprender el lenguaje corporal es tan importante como la comunicación verbal en las relaciones profesional sanitario-paciente	6.75	.563	.346**
5	El humor del profesional sanitario contribuye a obtener un mejor resultado en el tratamiento	6.11	.900	.441**
6	Como cada persona es diferente. es difícil ver las cosas desde la perspectiva de los pacientes	4.66	1.495	.471**
7	Prestar atención a las emociones de los pacientes no es importante durante la entrevista	6.83	.712	.413**
8	Prestar atención a las experiencias personales de los pacientes no influye en los resultados del tratamiento	6.55	.865	.389**
9	Los profesionales sanitarios deben intentar ponerse en el lugar de sus pacientes cuando les atienden	6.51	.807	.410**
10	Los pacientes valoran que el profesional sanitario entienda sus sentimientos. lo cual es terapéutico por sí mismo	6.52	.658	.504**
11	Las enfermedades de los pacientes solo se pueden curar mediante el tratamiento; por tanto. Los vínculos emocionales establecidos entre el profesional sanitario y sus pacientes no influyen de manera significativa en los resultados del tratamiento	6.38	.869	.409**
12	Preguntar a los pacientes sobre lo que ocurre en su vida personal no es útil para comprender sus quejas/dolencias físicas	6.59	.923	.392**
13	Los profesionales sanitarios deben tratar de comprender lo que pasa por la mente de sus pacientes. Prestando atención a su comunicación no verbal y a su lenguaje corporal	6.67	.650	.482**
14	Creo que no hay cabida para las emociones en el tratamiento de las enfermedades médicas	6.57	.973	.351**
15	La empatía es una habilidad terapéutica sin la cual el éxito del profesional sanitario estaría limitado	6.38	.786	.531**
16	La comprensión del profesional sanitario acerca del estado emocional de sus pacientes. Así como de sus familias es un factor importante en la relación profesional sanitario-paciente	6.63	.593	.629**
17	Los profesionales sanitarios deberían tratar de pensar como sus pacientes para prestar una mejor atención	5.98	.892	.469**
18	Los profesionales sanitarios no deberían permitirse verse influidos por los fuertes vínculos establecidos con sus pacientes y familiares	3.71	1.624	.330**
19	No disfruto leyendo literatura no sanitaria. de humanidades o arte	5.36	1.805	.482**
20	Considero que la empatía es un factor importante en el tratamiento del paciente	6.71	.584	.517**

Note: ** = p < .01

### Convergent validity results

Similar to Hojat et al. [5], in our study we used the first three components of the IRI as a criterion validity variable. The JSE authors did not use the anxiety component (in order to shorten the questionnaire and increase the response rate), arguing that this dimension had less interest in the doctor-patient relationship. Statistical analysis showed significant positive correlations between the JSE-HPS total and dimensions’ scores and the overall IRI scores, as well as positive correlations with most of IRI subscales (Table 5).

**Table 5:** Correlations between JSE-HPS and the Sensory Reactivity Index (IRI)

IRI	Dimension 1	Dimension 2 (FS)	Dimension 3 (EC)	Dimension 4 (PD)	IRI – total
JSE-HPS	(PT)				
Dimension 1 (PT)	.307**	.408**	.272**	−.030	.455**
Dimension 2 (CC)	.279**	.276**	.125	−.059	.304**
Dimension 3 (SPS)	.041	.068	.144*	−.157*	.153*
JSE-HPS – Total	.315**	.369**	.226**	−.071	.424**

Note: *PT* (Perspective Taking); *CC* (Compasionate Care); *SPS* (Standing in Patient’s Shoes); *FS* (Fantasy); *EC* (empathic Concern); *PD* (Personal Distress); * = *p* > 0,05; ** = *p* < 0,01

## Discussion

This study constitutes the first Spanish validation of the JSE-HPS scale in occupational therapy students. This internationally known scale has been used in numerous research studies to analyze the level of empathy of university students from different health professions, such as medicine, nursing, pharmacy, or physiotherapy [12, 42, 43].

The process of cultural adaptation carried out in this study followed a specific methodology, including being analyzed in detail by bilingual translators, in addition to having several differentiated phases of blinded analysis. Following this strict review process facilitate that the comprehensibility and grammatical stability of the translated version was adequate to facilitate the correct interpretation of each of the items on the scale. As well as in other questionnaires, the process of cultural adaptation ensure that the comprehensibility of the items formulated in negatives was understood without difficulty by the panel of experts, since these items are essential to minimize response bias and act by reducing the speed of response and favoring the respondent’s reasoning [44]. In addition, they contribute to the validity of the measure, making it easier for the subject to objectify how the construct under study is related to his or her beliefs [45]. Therefore, the resulting version guaranteed that the translated items were adjusted to the cultural context and allowed its use in Spanish occupational therapy educational and research contexts.

The sample size used in this study was large and, although the recommendations on required sample sizes in the CFA literature are all ad-hoc guesses [46] and even contradictory [47], existing recommendations and rules of thumb, such as the N > p or N:q ratio, which are commonly used for minimum recommendations, were considered in this study. In addition, other important aspects to consider during power analysis are the overall model fit and likelihood ratio tests [48–51], as well as the behavior of the Chi-square statistic, the RMSEA and other fit indices at different sample sizes. As it is suggested in literature [52] as well as in previous studies [53–56], the psychometric analysis of the JSE-HPS adapted version, was confirmed through CFA, determining the identification of three clearly differentiated empathic components and factorial loads greater than 0.30, which ensure an adequate scale dimensionality as observed in previous studies Hojat & LaNoue [57].

As in numerous investigations, the reliability of the scale was calculated using Cronbach’s alpha coefficient. In our study, the adapted version of the JSE-HPS scale showed adequate internal consistency with an α value of 0.786, which moves away from alpha values below 0.7 that indicate too high heterogeneity and does not reach values above 0.9 that are indicative of redundancy or duplicity of items. However, although the alpha value must be high to empirically demonstrate reliability, it should be taken into account that alpha is an index of internal consistency and does not provide information on the number of factors that explain the correlations of the items, and therefore it should be the FA that explains the structure of the observed correlations [58]. Moreover, as in previous studies conducted in similar cultural contexts [56], the intraclass correlation coefficients of the JSE-HPS-S obtained in this research showed adequate values between 0.77–0.93.

As observed in different studies and cultural contexts [43], the percentage of women who study occupational therapy is high. In this study, a percentage higher than 80% of the total sample of participants were women. According to our results, as well as the results obtained by Hojat et al. [11], women obtained significantly higher scores than men and were identified with higher scores in the first dimension analyzing the taking of perspective, which was considered a cognitive factor of empathy. These results, which reflect higher scores in females, have been observed in studies carried out in different countries and in researches that have used different versions [13, 14, 16, 59–61]. All this may be due to multiple factors (cultural, educational, biological, and even genetic) which have been widely discussed in other works, and which also suggest that the traditional and evolving role of women as caregivers and the ability to accept and integrate emotional aspects may be a factor responsible for this type of results [22–24, 62].

In previous studies, Mathad et al. [63] observed and analyzed the significant correlations between empathy and different aspects, such as emotional intelligence and resilience. These factors have been considered as characteristics that facilitate interpersonal relationships due to allowing one to be aware of, understand and manage emotions in oneself and others, and permit their use for better reasoning. In the current study, the use of the IRI, considered the gold standard test to analyze the multiple dimensions of empathy, has allowed us to observe the general dimensions studied in the JSE-HPS-S version. These findings show adequate and statistically significant results, similar to those obtained in other works [53, 54], guaranteeing that the measurement of the main construct of the resulting version is adequate, and providing adequate and similar data of convergent validity to those observed in previous studies [55].

Despite the fact that the results obtained are adequate, no statistically significant correlations were observed in three of the items belonging to the block of the patients’ perspective. This may be due to the fact that the scale was administered to students of the four courses of the degree of Occupational Therapy, of which the students of the first two courses had not experienced direct contact with the patients, and the students of the third course were starting their clinical practices in healthcare centers with different patient profiles. Therefore, insecurity, motivation or disposition before this first clinical contact may be one of the factors or selection biases responsible for these types of results.

The study has potential limitations. First, although the current study has followed a specific and precise process for its adaptation and validation phase, the sample corresponds to students from a single institution. Therefore, despite the sample being adequate, it would be advisable to expand the sample size and diversity of the surveyed students to facilitate the analysis and confirmation of its external validity.

Another aspect that may limit the results is the lack of clinical contact of some of the respondents, since they have not carried out clinical practices. The results, however, indicate that the differences are not significant, so exposure to patients may not be a differentiating factor. Whether sex was an influencing factor in these findings is worthy of future.

This study also provides some evidence that the JSE-HPS version is a valid and reliable scale in a cross-cultural context. Future research needs to examine whether this pattern would be repeated across occupational therapy students at other universities. Additional studies involving health professions are recommended, using larger sample.

## Conclusions

The Spanish version of the JSE-HPS scale has displayed good validity, indicating that it can be a useful instrument to assess empathy of occupational therapy students. Wide dissemination of the JSE-HPS scale and its different validated versions allow for the assessment of empathy in various settings and comparison of results with other studies in different populations. Having a culturally adapted and validated instrument to measure empathy will facilitate the evaluation of outcomes of occupational therapy training programs designed to develop empathy skills. However, more research is needed to examine empathy and analyze the factors that contribute to its development in both occupational therapy studies and professional practice.

## Data Availability

All data or analyzed during this study are included in this published article (and its supplementary information files). You can contact the correspondence author patricia.sanchezherrera@urjc.es to request all the necessary data and materials.
